# A simplified and high-yield purification method for recombinant RNase R

**DOI:** 10.17912/micropub.biology.002157

**Published:** 2026-06-24

**Authors:** Wataru Horikawa, Daniel L. Kiss

**Affiliations:** 1 Center for RNA Therapeutics, Houston, TX, United States; 2 Department of Cardiovascular Sciences, Houston, TX, United States; 3 Houston Methodist Academic Institute, Houston, TX, United States; 4 Houston Methodist Research Insititute, Houston, TX, United States; 5 Weill Cornell Medical College, Houston, TX, United States; 6 Houston Methodist Neal Cancer Center, Houston, TX, United States; 7 Houston Methodist Research Institute, Houston, TX, United States

## Abstract

RNase R is a 3’→5’ exoribonuclease that selectively degrades linear RNAs. As such, RNase R has become a key reagent for circRNA production and analysis. This is particularly true for the in vitro production of circRNA research tools and therapeutic candidates. Unfortunately, the high cost of commercial RNase R is restrictive. We report an accessible, cost-effective method to purify high-quality recombinant RNase R from E. coli using single-step Ni NTA chromatography on entry level FPLC systems. By achieving complete linear RNA digestion while maintaining circRNA, the resulting enzyme matches performance attributes of commercially available RNase R.

**Figure 1. Construction, purification, and qualification of recombinant RNase R:** (A) Schematic representation of His6 and V5-tagged recombinant RNase R (87-725). (B) Protein purification procedure for recombinant RNase R. (C) Elution chromatography of recombinant RNase R via an ÄKTA Start. (D) Image of a stain-free SDS-PAGE gel of recombinant RNase R elution fractions around elution peak shown in panel C. kDa indicates protein size. WCL: whole cell lysate. The asterisk marks the position of recombinant RNase R. (E) Purified recombinant RNase R in stock solution after dialysis. In vitro validation of RNase R activity using a (F) 5’ FAM-labeled RNA oligonucleotide and (G) a complex mix of linear and circular RNAs. RR is the RNase R purified here. For panels C - G one representative result from three or more independent purifications are shown. In panel F, the bars show mean ±SD of three independent experiments

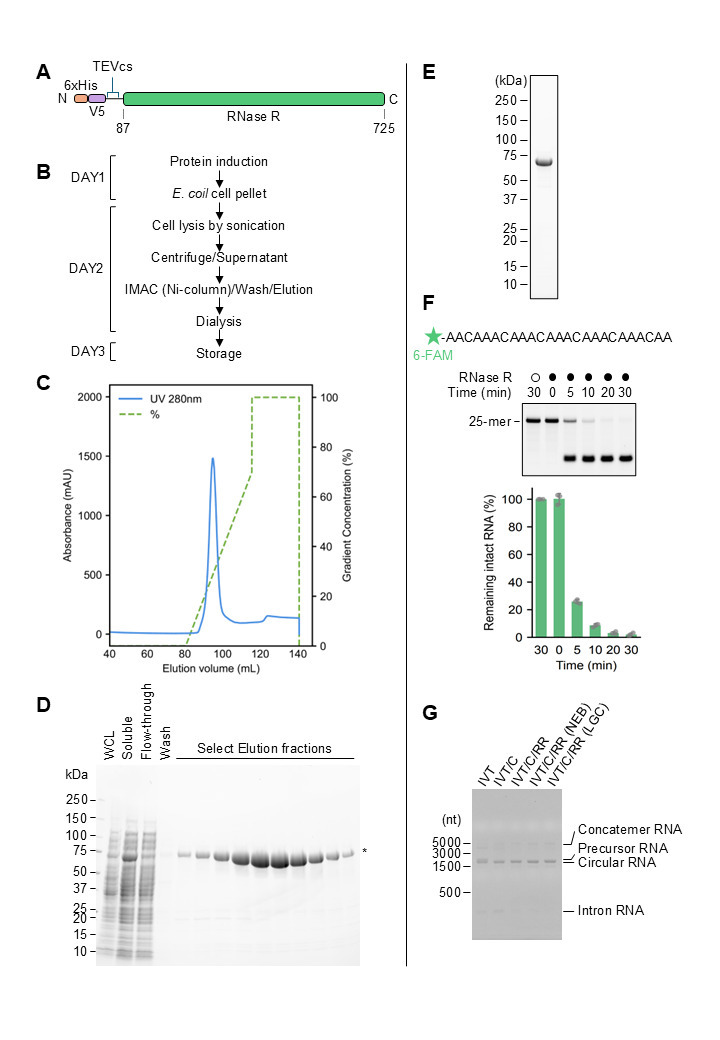



## Description

The past three decades have seen RNA Biology transform from a niche field into a dynamic study area with direct impact across science and human health. As research into RNA biology and RNA therapeutics has broadened, more tools are needed to further RNA research. The ability to degrade RNAs in a controlled and specific manner is a key tool to purify and/or test candidate RNA therapeutics.

Among exoribonucleases, RNase R, a 3'-to-5' exoribonuclease derived from the *Escherichia coli rnr* gene, occupies a unique niche. Belonging to the RNR superfamily, it can digest almost all linear RNA species while leaving circular RNA (circRNA) and lariat structures intact. Beyond its exoribonuclease activity, RNase R can unwind and degrade structured RNAs, including secondary structures that typically block other exonucleases (Cheng and Deutscher 2002, Hossain, Malhotra and Deutscher 2016, Chu, Hsieh et al. 2017). Furthermore, its specificity for RNAs with free 3' ends has made RNase R an indispensable tool for the enrichment and identification of circRNAs and the development of circRNA-based therapeutics and vaccines (Wesselhoeft, Kowalski and Anderson 2018, Wesselhoeft, Kowalski et al. 2019, Qin, Castillo et al. 2025).

Unfortunately, the high cost of commercially available RNase R remains a significant bottleneck for circRNA research. While several laboratory-scale purification protocols have been published, they involve multi-step chromatography and/or time-consuming tag-cleavage processes to achieve both high purity and high activity. Such steps increase cost and protocol complexity while potentially reducing yields.

A previous paper reported that deleting both the N- and C-terminal ends of RNase R, which are predicted to form unstructured regions, retained the activity of full-length RNase R (Chu, Hsieh et al. 2017). In general, eliminating unstructured regions makes recombinant proteins easier to purify by reducing nonspecific protein-protein interactions. Therefore, we use a truncated form (amino acids 87-725) of RNase R ( Figure 1A ) (Horikawa and Kiss 2026). Here, we describe a streamlined and efficient purification protocol ( Figure 1B ) for recombinant His-tagged RNase R. By optimizing the expression conditions and the affinity chromatography wash steps, the protocol yields ~40 mg of purified protein per liter of culture (Figures 1D and 1E). As the enzyme is highly active while the N-terminal His and V5 tags are retained, eliminating them by protease cleavage is not necessary.

The primary strength of this method is its efficiency and robust performance. By optimizing the reaction buffer and the enzyme-to-substrate ratio, we have established a purification and reaction system that yields RNase R indistinguishable from vendor-purchased RNase R (Figures 1F and 1G). This optimized framework ensures the complete and specific degradation of linear RNA, providing high-quality circular RNA enrichment ( Figure 1G ) suitable for sensitive downstream applications with circRNA. Given its high yield and simplified workflow, this method offers a highly accessible and reliable alternative for the global RNA research community. Please note, the RNase R purified via this protocol may contain some endotoxin that may need to be removed if treated/digested RNAs will be transfected into cells.

We describe the procedures for expressing, purifying, and ensuring the solubility of active RNase R. However, protein yields will likely vary based on several factors including the type of medium employed, the size of cell pellets at the start points and the condition and quality of the transformed cells. As such, we advise using freshly transformed cells. In addition, we recommend expediting the protein purification process to avoid protein degradation. In addition to these suggestions, several additional parameters deserve special consideration. We found that using high quality LB broth was critical to achieving high yields of recombinant RNase R. We also found that adding 2-Mercaptoethanol as a reducing reagent to lysis and wash buffers is important for eliminating nonspecific protein binding during column loading and washing steps. However, we also advise users to remove reducing reagents from the elution and dialysis buffers. This enables direct protein concentration measurements using a spectrophotometer.

With regards to assaying RNase R activity and using it as a tool in the lab, we also highly recommend that you use optimized buffer described in the methods below. We observed that using commercially available RNase R reactions buffers lowered RNA degradation efficiency when used with RNase R purified by this protocol. RNase R stored at -20°C for over 1 year still possesses robust RNA degradation activity (Horikawa and Kiss 2026). Lastly, we also used this protocol to purify an inactive forms of RNase R; however, our preparations were always contaminated with *E coli* nucleic acids, suggesting that the D280N mutant was effectively a ‘substrate trap’ that irreversibly bound RNAs (Horikawa and Kiss 2026).

As detailed above, this method has been optimized to produce highly active RNase R for use in laboratory preparations and experiments where eliminating linear RNA species is a key goal. While we show that the purified RNase R rapidly degrades linear RNAs while leaving circular RNAs intact (Figures 1F and 1G), we have not tested this truncated (and tag-retaining) form of RNase R in mechanistic studies aimed at better understanding RNase R's enzymatic activity. This could be a concern since mechanistic studies may be impeded by this protocol's retention of the highly charged 6xHIS and/or V5 tags at the protein's N-terminus. Furthermore, the N- and C-terminal truncations that facilitate the purification of RNase R-87-725 could also complicate some mechanistic studies. Indeed, supporting that thought, an N- and C-terminally-truncated D280N mutant RNase R (RNase R-87-725-D280N) is incapable of binding RNA, while an N-terminally-intact (but C-terminally truncacted) D280N mutant RNase R (RNase R-1-725-D280N) is capable of binding -albeit inefficiently- RNA (Horikawa and Kiss 2026). For these reasons, we suggest that users whose aim is to understand the mechanism and/or kinetics of RNase R activity should either utilize the TEV cleavage site during protein purification, obtain and purify the protein by using the full-length expression constructs (Addgene #254214, #254215), or both (Horikawa and Kiss 2026).

## Methods

Please note, the highly detailed step-by-step purification protocol is available on bioRxive (Horikawa and Kiss 2026).

Chemically competent BL21 Star (DE3) *E. coli* were transformed with a plasmid encoding RNase R (Addgene #254213), plated onto LB ampicillin plates and allowed to grow at 37°C overnight. Colonies were used to inoculate a 100 ml starter culture (LB medium with 50 µg/ml Ampicillin) which was incubated at 37°C with shaking at 220 rpm overnight. 10 ml of the overnight culture was added to 1 L of LB with 50 µg/ml Ampicillin and grown at 37°C with shaking at 200 rpm until OD _600_ reaches 0.3 to 0.4. The temperature was then reduced to 18°C and cultures were grown for 1 hr at 18°C with shaking at 200 rpm. Expression of RNase R was induced by adding IPTG to a final concentration of 0.5 mM and cultures were incubated overnight at 18°C with shaking at 200 rpm.

Cultures were pelleted by centrifugation for 10 min at 5000 x *g* , 4°C and the pellets (~4-5 ml volume) were transferred to a 50 ml conical tube. Ice cold lysis buffer ***(*** *50 mM Tris, pH 8.0, 1 M NaCl, 30 mM Imidazole, 0.3% (w/v) Tween 20, 5 mM 2-Mercaptoethanol)* was added to the tube until the buffer reaches 30 ml and pellets were resuspended by vortexing. Cells were lysed by sonication, and insoluble cellular debris was removed by centrifugation (45 min at 30,000 x *g* , 4°C). The cleared lysate was loaded onto a pre-equilibrated HisTrap HP 5 mL column using a 3 ml/min flow rate. Unbound proteins were removed with 16 column volumes (80 ml) of wash buffer ***(*** *50 mM Tris, pH 8.0, 300 mM NaCl, 30 mM Imidazole, 5 mM 2-Mercaptoethanol* ***).*** Fractions were collected and absorbance at 280 nm ( Figure 1C ) was measured as the protein was eluted with a linear gradient increasing to 70% elution buffer ***(*** *50 mM Tris, pH 8.0, 300 mM NaCl, 500 mM Imidazole* ***)*** 7 column volumes and then 100% elution buffer for 5 column volumes.

Purified proteins were assessed by SDS PAGE using stain-free gels. The proteins were visualized using a ChemiDoc Imaging System on Stain Free Gel mode (Figures 1D, 1E). Protein containing fractions were pooled and dialyzed overnight in 1L of Dialysis buffer ***(*** *50 mM Tris, pH 8.0, 300 mM NaCl, 50% (v/v) Glycerol) at* 4°C with slow stirring. Protein concentration was calculated using a spectrophotometer and an extinction coefficient of 63,260/M/cm. The remaining protein was aliquoted and stored at -20°C.

RNase R activity was measured using a 5’-6-FAM-labeled 25-mer RNA oligonucleotide [FAM]-AACAAACAAACAAACAAACAAACAA ( Figure 1F ). 100 nM of oligonucleotide was incubated with 100 nM recombinant RNase R in reaction buffer (20 mM Tris-HCl (pH 7.5), 100 mM KCl, 0.25 mM MgCl _2_ ) at 37°C for the indicated times in 20 µl reactions. Reactions were quenched by adding 60 µl of RNA Loading Dye (95% (v/v) Formamide, 17.5 mM EDTA, 0.01% Bromophenol Blue) followed by running 7 M Urea/20% polyacrylamide gel in 1x TBE buffer. Signal intensities of intact RNA were quantified by Fiji software. RNase R’s activity and specificity for linear RNAs were evaluated by comparing its activity to RNase R’s obtained from two different vendors using an in vitro transcribed circular RNA with known linear processing byproducts ( Figure 1G ). RNA was incubated with the indicated RNase R for 30 min at 37°C in 1x RNase R reaction buffer and reaction buffers provided by venders. 0.3 µg recombinant RNase R and 1 U commercial RNase R were utilized per 1 µg RNA. RNA was extracted using RNA Clean & Concentrator-5 according to the manual and 100 ng RNA was used for running the gel. All three RNase Rs degraded linear RNAs, but left circRNA intact ( Figure 1G ).

## Reagents

**Table:** 

Reagent	catalog number
Plasmid DNA encoding E. coli Rnase R	available via Addgene plasmid repository
One Shot BL21 Star (DE3) Chemically Competent Cells	Invitrogen, REF: 44-0049
LB agar (1.2%) plate with appropriate antibiotics	BD, REF: 240230; Apex Bioresearch, Cat#: 20-273
Ampicillin, 100 mg/ml	Sigma-Aldrich, Cat#: A5354-10ML
SOC Outgrowth Medium	New England Biolabs, Cat#: B9020S
Antifoam 204	Sigma-Aldrich, Cat#: A6426
HisTrap HP 5 mL	Cytiva, Cat#: 17524802
2x Laemmli Sample Buffer	Bio-Rad, Cat#: 1610737
Precision Plus Protein Unstained Standards	Bio-Rad, Cat#: 1610363
Mini-PROTEAN TGX Stain-Free Precast Gels, 4-20%, 15-well comb	Bio-Rad, Cat#: 4568096
Spectra/Por 6 Dialysis Membrane Pre-wetted RC Tubing, MWCO: 3.5 kD	Spectrum, REF: 132592
Spectra/Por Closures Standard Type, 55 mm	Spectrum, REF: 132737
Spectra/Por Closures Weighted, 55 mm	Spectrum, REF: 132745
Slide-A-Lyzer Buoys	Thermo Scientific, REF: 66432
MCE Membrane Filter, 0.22 ?m Pore Size	Millipore, REF: GSWP04700
IPTG (Isopropyl-?-D-thiogalactopyranoside), Dioxane Free	RPI, Cat#: I56000
LB medium (Lennox)	BD, REF: 240230
Imidazole	Thermo Scientific, Cat#: A10221.36
Tris (1 M), pH 8.0	Invitrogen, REF: AM9856
5 M NaCl	Invitrogen, REF: AM9759
2-Mercaptoethanol	Sigma-Aldrich, Cat#: 63689-100ML-F
Glycerol, ACS Grade	RPI, Cat#: G22025
Tween 20	Sigma-Aldrich, Cat#: P9416-100ML
[FAM]-AACAAACAAACAAACAAACAAACAA	IDT
